# Severe Overt Hypothyroidism-Induced Rhabdomyolysis Complicated by Acute Renal Impairment

**DOI:** 10.7759/cureus.71996

**Published:** 2024-10-21

**Authors:** Jeremy A Knott, Thaw Htet

**Affiliations:** 1 Endocrinology, St George Hospital, Sydney, AUS; 2 St George and Sutherland Clinical School, University of New South Wales, Sydney, AUS

**Keywords:** creatine kinase, hashimoto's hypothyroidism, hypothyroid-induced rhabomyolysis, medication non-compliance, renal impairment

## Abstract

Musculoskeletal symptoms in hypothyroidism are often vague and non-specific, but in rare cases, rhabdomyolysis may develop as a serious complication. Here, we report a case of a 25-year-old man with a known history of Hashimoto's thyroiditis who presented with symptoms of rhabdomyolysis complicated by renal impairment secondary to severe overt hypothyroidism in the context of medication non-compliance. He presented with symptoms of generalised myalgia and fatigue. Laboratory investigations were consistent with severe overt hypothyroidism with thyroid-stimulating hormone (TSH) 531.4 mIU/L and free thyroxine (T4) 0.9 pmol/L (0.07 ng/dL). Creatine kinase (CK) levels were elevated at 1052 U/L with associated acute renal impairment, creatinine 129 *μ*mol/L (1.49 mg/dL). Our patient was managed with the recommencement of thyroxine therapy and intravenous hydration. Over the course of hospitalisation, the patient's myalgias gradually improved, with an improvement in CK levels and renal function. Our case highlights the potential consequences of prolonged non-compliance. Clinicians should remain vigilant in monitoring patients' medication adherence and be aware of the possible complications from non-compliance. Early recognition and prompt management of such cases can lead to successful recovery and prevent long-term sequelae.

## Introduction

Thyroid disorders are prevalent in the general population affecting approximately 5-10%, with a higher incidence of hypothyroidism compared to hyperthyroidism [1]. Clinical symptoms of hypothyroidism are often non-specific including muscular manifestations, which can vary from myalgia, fatigue, and cramping, occurring in up to 70% of cases [2]. Elevations of serum creatine kinase (CK) may occur in hypothyroid patients; however, hypothyroidism-induced rhabdomyolysis, with rapid skeletal muscle breakdown, is rare [3].

Literature reviewing hypothyroid neuromuscular complications are predominately based on retrospective analyses often involving selected case series, and interestingly appear to occur more commonly in the ma;e gender [4], despite hypothyroidism being more common in women, with reported cases often identifying precipitating factors, including strenuous exercise [3-5] or use of statin therapy [6]. We present a case of a 25-year-old male patient with a known history of Hashimoto’s hypothyroidism and prolonged thyroxine non-compliance, who presented with new onset rhabdomyolysis and acute renal impairment.

This article was previously presented as an abstract poster presentation at the Endocrine Society of Australia 2023 Annual Scientific Meeting on November 27, 2023 [7].

## Case presentation

A 25-year-old Caucasian man presented with a five-month duration of severe generalised myalgia and associated intermittent muscular stiffness and cramping affecting his entire body. He also reported a progressive history of generalised lethargy, unintentional weight gain of 5 kgs, cold intolerance, constipation, low mood and dry skin during that time. He denied any trauma, strenuous exercise, statin therapy, anabolic steroids, alcohol or use of creatine. He was diagnosed with Hashimoto's thyroiditis at age 15, with a significant autoimmune family history of Hashimoto’s in his mother and sister, as well as depression on regular escitalopram. The patient reported non-compliance with his previously prescribed thyroxine 200 mcg daily dose for the past year due to miscommunication regarding medication instructions as well as missing regular follow-up appointments.

Physical examination revealed an elevated BMI of 31 kg/m² with central obesity. He was afebrile with a regular heart rate of 62 beats per minute and normotensive at 109/78 mmHg. He had mild facial puffiness, dry skin over his hands and a diffuse, non-tender thyroid gland that was not enlarged. There was no evidence of muscle wasting or muscle weakness. There were no other features suggesting overt hypothyroidism, including alopecia, brittle hair, coarse facies, hoarse voice, bradycardia, or delayed relaxation of deep tendon reflexes. He was able to mobilise independently.

**Figure 1:**
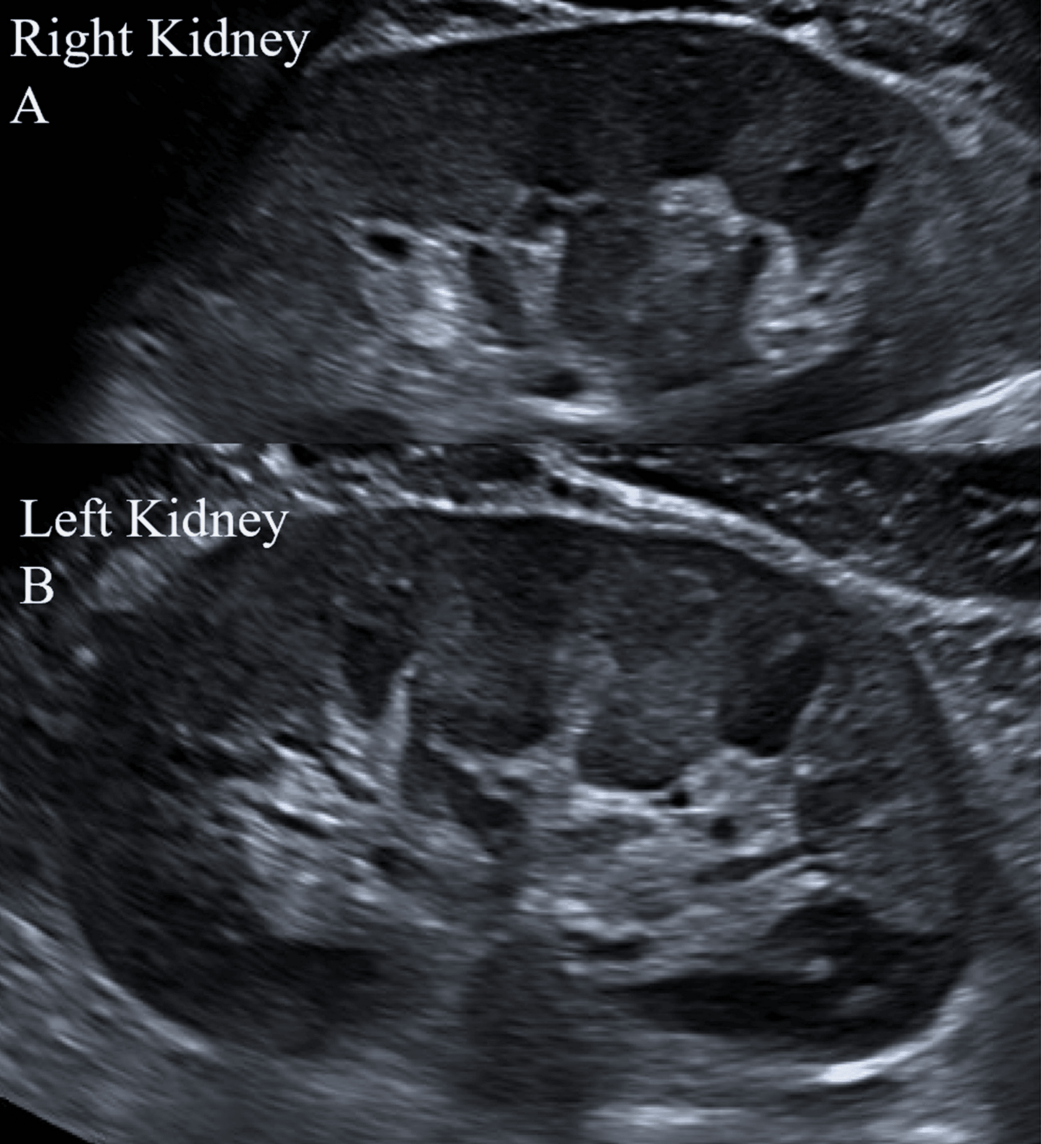
Renal tract ultrasound revealed no structural abnormalities in the right (A) and left (B) kidneys.

Laboratory investigations revealed severe overt hypothyroidism with thyroid-stimulating hormone (TSH) at 531.4 mIU/L and free thyroxine (T4) at 0.9 pmol/L (Table 1). His autoantibodies were elevated; thyroid peroxidase antibody >600 IU/mL and thyroglobulin antibody 1408 IU/mL. There was marked elevation in creatine kinase (CK) 1052 U/L with associated acute renal impairment with a raised creatinine (Cr) 129 μmol/L from baseline 94 umol/L. Urinalysis revealed microscopic haematuria consistent with myoglobinuria. Urine drug screen was negative. Imaging with a renal tract ultrasound revealed no structural abnormalities (Figure 1). The rest of his electrolyte panel including sodium, potassium, corrected calcium, phosphate, and magnesium levels were within normal limits. His 25-hydroxy vitamin D was borderline deficient at 48 nmol/L.

**Table 1:** Initial biochemistry results

Investigations	Results	Reference Range
Thyroid-stimulating hormone	531.4 mIU/L	0.27 – 4.2 mIU/L
Free-thyroxine	0.9 pmol/L	12 – 22 pmol/L
Thyroid peroxidase antibody	>600 IU/mL	0 – 34 IU/mL
Thyroglobulin antibody	1408 IU/mL	0 – 115 IU/mL
Creatine kinase	1052 U/L	45 – 250 U/L
Creatinine	129 *μ*mol/L	60 – 100 *μ*mol/L
Sodium	138 mmol/L	135 – 145 mmol/L
Potassium	4.1 mmol/L	3.6 – 5 mmol/L
Corrected calcium	2.23 mmol/L	2.15-2.55 mmol/L
25-hydroxy vitamin D	48 nmol/L	>50 nmol/L
Phosphate	1.21 mmol/L	0.75 – 1.5 mmol/L
Magnesium	0.83 mmol/L	0.7 – 1.1 mmol/L
Urine drug screen	Negative	-

**Figure 2:**
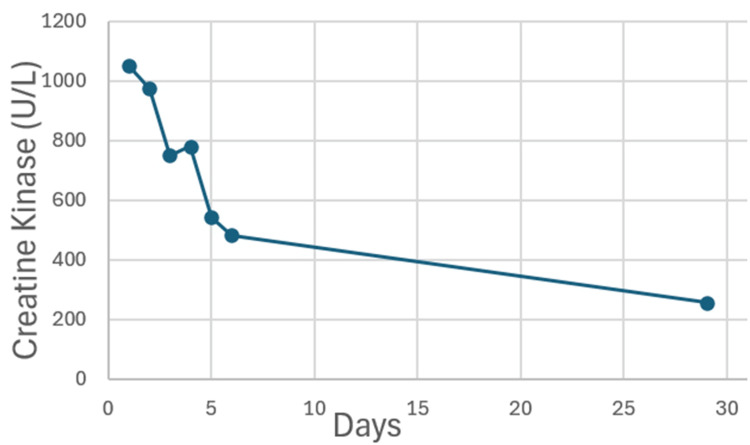
Graph depicting improved creatine kinase levels with thyroxine and intravenous fluid therapy

The patient was managed with intravenous fluid hydration and recommenced on thyroxine therapy 150 mcg daily. After six days of intravenous hydration, there was gradual improvement in renal function (Cr 118 μmol/L) and reduction in CK to 482 U/L as well as a clinical improvement in his muscle stiffness and intermittent spasms. He was also educated regarding the appropriate timing of taking thyroid medication, strict compliance and regular monitoring. On the three-week follow-up, his renal function remained elevated at Cr 119 μmol/L compared to the previous baseline with continued improvement in serum CK at 256 U/L (Figure 2).

## Discussion

Hypothyroidism presents with a diverse range of non-specific clinical manifestations, as demonstrated in our patient including constipation, dry skin, weight gain, and depressive symptoms. However, musculoskeletal symptoms are often limited to muscle cramps, stiffness, myalgias, and generalised fatigue, with an occasional associated mild elevation of muscle enzymes [8]. Rhabdomyolysis is an uncommon presentation, characterised by the necrotic breakdown of skeletal muscle and release of intracellular muscle contents into circulation, and can stem from various causes including intense physical exercise, trauma, congenital and inflammatory myopathies, infections, electrolyte disorders, medications such as statins, hyperosmolar states, and toxins such as alcohol. Hypothyroidism-induced rhabdomyolysis, in the absence of a clear precipitant or non­compliance with therapy, as in our case, is even rarer [8].

Hypothyroidism-induced myolysis is attributed to a reduction in muscle cell mitochondrial activity and dysregulation of metabolic pathways including glycolytic energy production and fatty acid catabolism necessary for muscle cell function. This leads to altered expression of contractile proteins with reduced myosin ATPase activity as well as increased glycosaminoglycan polysaccharide deposition in skeletal muscle and neuro-mediated damage [9]. This causes increased atrophy of fast-twitch type II muscle fibres, due to their dependency on glycolysis for energy supply, with a compensatory hypertrophy of slow-twitch type I fibres. These metabolic changes may sensitise hypothyroid patients to prolonged oxidative damage, which may increase the susceptibility to develop rhabdomyolysis, especially when coupled with other precipitating factors [5].

Acute renal impairment is a serious complication and can develop in up to 33% of patients with rhabdomyolysis [10], as occurred in our patient. Renal dysfunction due to hypothyroid-induced rhabdomyolysis has been rarely reported. The release of musculoskeletal intracellular components, including myoglobin, into circulation can impact renal filtration and lead to acute tubular necrosis and subsequent renal impairment. Hypothyroidism itself may further impair renal blood flow, due to haemodynamic effects on decreasing cardiac output and increasing renal and systemic vascular resistance [11]. Intravenous hydration and thyroxine replacement should be promptly instigated to prevent further muscle breakdown.

Our patient presented with clinical and biochemical findings of rhabdomyolysis complicated by renal impairment due to severe overt hypothyroidism in the context of thyroxine non-compliance for one year prior to admission with no other precipitating factors. Increased clinical awareness of this rare complication may modify clinical practice by highlighting the importance of strict medication compliance through patient education and providing a duty of care to ensure regular monitoring of thyroid function. Employing communication language techniques such as the 'teach-back method' can help ensure patient understanding and prevent miscommunication [12]. Our case emphasises the importance of advising patients with thyroid disorders to exercise caution with potential rhabdomyolysis triggers, such as avoiding alcohol consumption, and the need for screening for thyroid dysfunction before prescribing other precipitating factors such as statin therapy.

## Conclusions

Severe overt hypothyroidism-induced rhabdomyolysis with secondary renal impairment are rare but potentially life-threatening complications. Clinicians should ensure appropriate patient education and remain vigilant in monitoring patients' adherence to thyroxine replacement therapy and promptly address non-compliance. Early recognition and management of rhabdomyolysis is crucial for ensuring successful recovery and preventing long-term sequelae.
